# Enhanced spring warming in a Mediterranean mountain by atmospheric circulation

**DOI:** 10.1038/s41598-022-11837-x

**Published:** 2022-05-11

**Authors:** E. Bruley, F. Mouillot, T. Lauvaux, S. Rambal

**Affiliations:** 1 Centre d’Ecologie Fonctionnelle et Evolutive CEFE, UMR5175, CNRS, Université de Montpellier, Université Paul-Valéry Montpellier, EPHE, 1919 Route de Mende, 34293 Montpellier Cedex 5, France; 2 Centre d’Ecologie Fonctionnelle et Evolutive CEFE, UMR 5175, CNRS, Université de Montpellier, Université Paul-Valéry Montpellier, EPHE, IRD, 1919 Route de Mende, 34293 Montpellier Cedex 5, France; 3 Laboratoire d’Ecologie Alpine, CNRS, Université Grenoble Alpes, Grenoble, France; 4 Laboratoire des Sciences du Climat et de l’Environnement, IPSL, Université de Saclay, Saclay, France; 5 Departamento de Biologia, Universidade Federal de Lavras, CP 3037, Lavras, MG CEP 37200-000 Brazil

**Keywords:** Climate-change ecology, Climate sciences, Forest ecology

## Abstract

We analyzed trends of air temperature across the Cévennes National Park in Southern France, a mid-altitude coastal mountain experiencing a rapid spread of forests at the expense of rangelands and submitted to Mediterranean Sea influences and so, impacted by local and regional processes of climate change. Since 1980, April to June warming trend reached a maximum temperature increase of + 0.124 °C year^−1^ and uniform whatever the altitude. Minimum temperature increased by + 0.058 °C year^−1^ at 500 m altitude and + 0.089 °C year^−1^ at 1500 m. Concomitantly, forest cover is increasing by + 0.51% year^−1^. Using an intrinsic biophysical mechanism model, we demonstrated that, at monthly scale, the forest surface is 1.7–3.1 °C cooler than that of nearby grasslands. As a result, the decrease in albedo corresponding to the conversion from grasslands to dense forests, translates into a cooling of maximum air temperatures of 0.023 °C year^−1^ which contributes to slow down the warming rate enhancement. Spring warming trends co-varied with negative WeMO phases associated with a low in the Gulf of Cádiz and an anticyclone in Central Europe. An east to west pressure gradient increases atmospheric humidity leading to a strong water vapor feedback, enhancing the forcing of thermal long wave radiations and hence the rise in temperature.

## Introduction

Variation in mean global surface air temperature is a key indicator of climate change. Evaluation of global warming rate is a source of controversies concerning (1) the nature of the data sources^1^ but also (2) the date from which the warming rate is estimated, i.e. the change point^2^ and finally, (3) the accounting or not of the "global warming hiatus"^3^. In addition, this global indicator provides no information on the spatial variation of the warming rate, e.g. zonal land vs. zonal ocean^4^ or on its seasonal variation^5^.

The enhancement of warming rates with elevation, the so-called elevation-dependent warming, is one of the regional, still not completely understood, expressions of global warming. Most studies based on observations indicate that warming rates are amplified with elevation, depending on season, study area and analyzed variable (usually minimum and/or maximum air temperature), but some other studies also show no clear trend with elevation^6–10^. Available observations suggest that Mediterranean mountains are experiencing seasonal warming rates that are largely greater than the global land average^11–13^. There is also evidence from field observations for elevation-dependent climate responses^14–16^.

Our understanding of climate change in mountains, however, remains challenging owing to inadequacies in observations and models^17,18^. In fact, it is still uncertain whether mountainous regions generally are warming at a different rate than the rest of the global land surface, or whether elevation-based sensitivities in warming rates are prevalent within mountains. To address questions related to the sensitivity of climate change to elevation, we need to explore processes that could lead to enhanced warming and possible mechanisms that can produce altitudinal gradients in warming rates on contrasted time scales. Mediterranean mountains bear a number of challenges for understanding, quantifying and simulating their land–atmosphere exchange of mass, energy and momentum. First, mountain areas are characterized by rapid changes in ecosystem structure and functions driven by changes in climate and land use along with elevation. A rapid spreading of forests and woodlands at the expense of rangelands is observed in all the Northern perimediterranean mountains^19,20^ and forest microclimate due to changes in canopy structure recently appeared as way more variable than macroclimate changes^21^. Second, their topographic features induce modifications to atmospheric flows and exchange processes which preclude theoretical frameworks developed for horizontally flat homogeneous terrain to be applied with confidence^6^. Third, the local near-surface atmospheric flows strongly influence the mesoscale circulation thus further complicating the use of common up- and downscaling approaches^22^. The resulting uncertainties limit our ability to project land–atmosphere interactions in mountain areas under changing future climate and land use^23^.

In addition to these local scale effects, regional atmospheric circulation patterns can be altered with global warming, in turn modifying mass flows at terrestrial/oceanic interfaces. Variations in sea-level pressure dipoles have been shown to influence climate northeast of the Iberian peninsula^24^ or to reduce and enhance water-limited areas influences on Mediterranean coastal areas^25^. They are also key factors affecting local climates, independently of the general warming environment.

In this work we focus on the Cévennes National Park in Southern France as a study case, combining a mid-altitude coastal mountainous situation in the Mediterranean hot spot of climate change, affected by land cover changes and at the interface between Atlantic and Mediterranean influences. As such, it is submitted to multifactorial impacts of local and regional scale processes of climate change. We specifically address the following questions:

1. In term of strength and seasonality, what are the ongoing elevation-dependent warming (EDW) rates for both minimal and maximal air temperature we observed over the whole study area?
2. Do land use changes contribute to accelerate or to slow down the enhancement of warming rates?
3. How do large-scale climate drivers indirectly influence strength and seasonality of EDW rates?

## Results

### Trends in monthly temperatures

**Figure 1:**
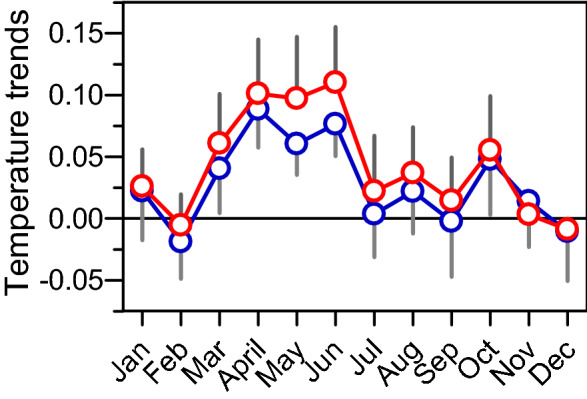
Linear trends in monthly maximum (red circle ± 95% CI) and minimum (blue circle ± 95% CI) temperatures for Mont Aigoual station over 1980–2014 (°C year^−1^). Significant trends were observed in April, May and June.

For the period considered, i.e. from 1980 to 2015, we calculated for each month and for all stations, the least-square linear trend of monthly average maximum temperatures (t_x_) and minimum temperatures (t_n_). Figure 1 plots the trends obtained for the Mont Aigoual station. Only trends in April, May and June were significant. For example, for t_x_ the June one reached 0.110 (95% confidence intervals 0.026, 0.029) °C year^−1^ or 1.1 °C per decade. For April, these trends reached 0.100 (0.025, 0.028) °C year^−1^ and 0.088 (0.027, 0.030) °C year^−1^ for t_x_ and t_n_ respectively. This pattern of change was observable for all stations. As a result, we aggregated months from April to June and analyzed the average temperatures over this period. For Mont Aigoual, t_x_ and t_n_ trends were then 0.103 (0.041, 0.044) °C year^−1^ (p < 0.001) and 0.076 (0.031, 0.040) °C year^−1^ (p < 0.001). Over the complete series of observations available for this station since 1901, Fig. 2 shows that the starting point of our analysis, i.e. 1980, appeared to be in the change point region detected by^26^.

**Figure 2:**
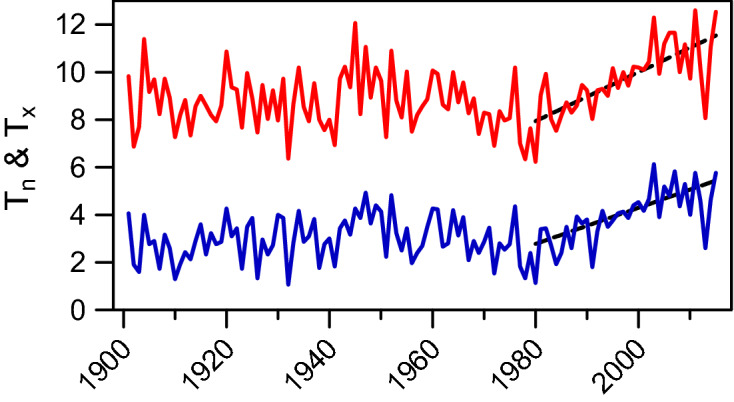
Time courses of mean maximal (red line) and minimal (blue line) April to June temperatures at Mont Aigoual station since 1901 (°C). The warming trend analysis has been done from 1980 (dashed black lines).

**Figure 3:**
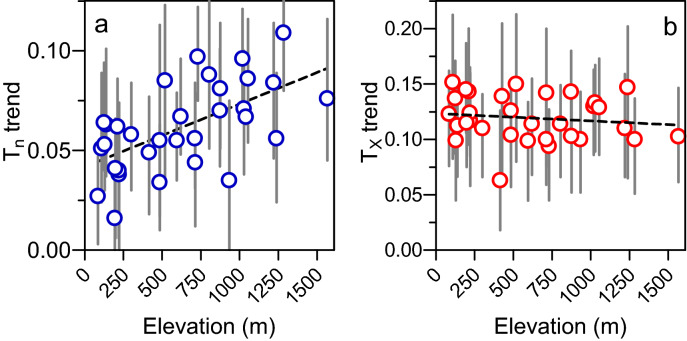
Linear warming trends observed on (**a**) mean minimal (blue circle ± 95% CI) and (**b**) maximal (red circle ± 95% CI) April to June temperatures (°C year^−1^) and elevation (m asl) for the 32 studied stations.

These significant warming trends were not exclusive to the highest station. Larger values were observed, for example, at Nasbinal and Brenoux-Mende, with 0.100 (0.049, 0.037) and 0.109 (0.029, 0.024) °C year^−1^ for t_x_ and 0.130 (0.044, 0.033) and 0.096 (0.026, 0.025) °C year^−1^ for t_n_ respectively. These stations are located at altitudes of 1284 m and 1019 m respectively. One low elevation station did not have a significant trend for their t_n_: Arphy. Altitude played a highly significant role in t_n_ trend (Fig. 3a), which increased linearly with a slope of 0.0032 ± 0.0008 °C year^−1^ per 100 m and a y-intercept of 0.0418 ± 0.0057 °C year^−1^ (R^2^ = 0.36; p-value = 0.0003). For t_x,_ no significant trends were observed (Fig. 3b). We can consider this trend as spatially uniform and equal to 0.124 ± 0.007 °C year^−1^, corresponding to a + 1.24 °C warming per decade. The effect of altitude was also tested by separating the stations according to their aspects. For t_n_ and t_x_, the slopes and intercepts were not significantly different depending on whether the aspect was either north-facing or south-facing slopes.

The long monthly precipitation time series available, 1901–2014, for 13 of the 32 stations do not show significant trends for annual totals or seasonal ones from April to June, either for the whole period or only for the period ranging from 1980 to 2014. Graphs for the wettest station, Mont Aigoual (mean annual precipitation MAP = 1792 ± 462 mm) and the driest one, Générargues (MAP = 1217 ± 331 mm) are reported in Fig SI3. For these two stations we analyzed the co-variation between rain totals from April to June and the corresponding t_x_ and t_n_. There is no significant effect of the precipitation amount on t_n_. For t_x_ the effect is noticeable, but not significant at 5% threshold. For Mont Aigoual and Générargues, we observed declines of t_x_ of 0.24 ± 0.15 °C (p-value = 0.11) and 0.48 ± 0.27 °C (p-value = 0.08) per 100 mm of precipitation. In 2008, the wettest year from 1980 to 2014, April to June precipitation was 907.8 mm at Mont Aigoual and 445 mm at Générargues. They caused theoretical declines in t_x_ of 2.2 °C and 2.1 °C respectively. The observed t_x_ were 10 °C and 22.3 °C for both stations.

### Vegetation maps and land use/land cover changes

Analyzing available maps, a significant and linear increase in the area of forests was observed, varying from 38% in 1955 to 68% in 2015 with a growth rate of 0.51 ± 0.08% per year (Table 1). This forest expansion occurred at the expense of non-forest areas, which declined from 62 to 25% over the same period with a rate of − 0.61 ± 0.09% per year. For all available maps, the transition class was not well assessed. Absent in the 1955 map, the percentage of transition zones reached 7% in 2015. Its growth rate was only 0.11 ± 0.05% per year.

**Table 1:** Time course of area of three major land cover types in the central protected zone of the Cévennes National Park.

Source	NGI	CNP	NFI	CNP	CLC	CLC	GISLR	CNP
Date	1955	1970	1999	2000	2000	2006	2006	2010
Forest	34,551	40,417	61,503	52,548	54,854	55,233	62,092	56,630
Open	56,850	47,757	28,993	35,157	29,014	31,188	19,974	29,943
Transition		2403	994	3512	7373	4902	9248	4722
Total	91,401	90,577	91,490	91,217	91,241	91,323	91,314	91,295

The sources are: *NGI* National Geographic Institute, *CNP* Cévennes National Park, *NFI* National Forest Inventory, *CLC* Corine Land Cover, *GISLR* Languedoc-Roussillon region.

For maps from the same source, i.e. CLC2000 and CLC2006, within the forest land cover type, broad leaved forests declined from 40 to 38%. Conversely, coniferous forests increased from 34 to 36%. This trend was ongoing until 2010 (CNP2010) since only 34% of broad leaved forests were present and coniferous forests reached 40%. Over the period 2000–2010, mixed forests accounted for a constant 26 percent value.

### Albedo

**Figure 4:**
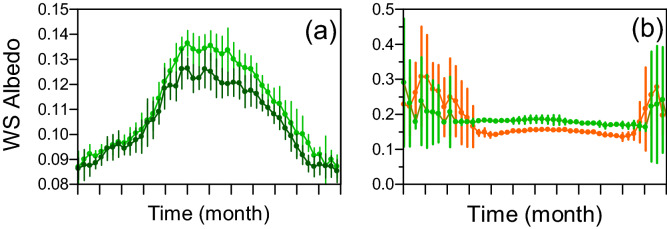
Twelve years (2000–2011) of 8-day mean ± SD MODIS white-sky albedo over (**a**) needle leafed forests (dark green) and broadleaf forests (light green) and (**b**) low shrublands (brown) and natural grasslands (light green).

**Figure 5:**
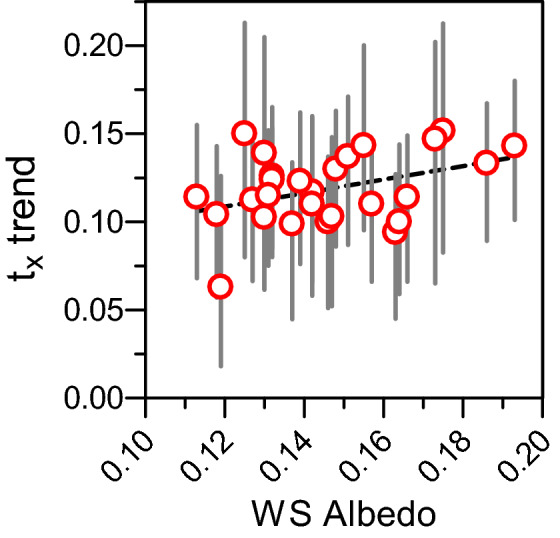
Linear relationship between WS albedo of area surrounding the weather stations and their warming trends for mean maximal temperatures (red circle ± 95% CI) from April to June, expressed in °C year^−1^.

Figure 4a and b present the within-year patterns of change in white sky albedo WSA for the four target areas: coniferous and broadleaf forest, low shrubland and natural grassland. These patterns cover the twelve years (2000–2011) of 8-day mean ± SD. For the two forested areas, winter albedos were very close with 0.085 ± 0.005 for the coniferous canopy and 0.087 ± 0.006 for the broadleaved one. Then albedo increased to peak early May at 0.126 ± 0.007 and 0.136 ± 0.005 followed by slight summer declines and then finally went back to winter values (Fig. 4a). The WSA temporal patterns for low shrublands and natural grasslands were affected by snow cover within the cold period. On average, snow began late November-early December for the two vegetation types and disappeared end of February in grasslands and later, end of March, in the low shrublands (Fig. 4b). During the vegetative period, albedos reached their maximum values at the beginning of July for natural grasslands (0.186 ± 0.011) and then mid-July for low shrublands (0.156 ± 0.005). Standard deviation was higher in natural grasslands because they were more sensitive to local variations of soil water limitations and erratic stormy rainfall. When pooling the three forest types in the forest land cover type, a significant linear relationship was observed between April to June WSA and percent forest cover (PFC) surrounding the 32 weather stations: WSA = − 7 × 10^–4^ ± 7 × 10^–5^ PFC + 0.186 ± 0.005 (R^2^ = 0.75, p-value < 0.0001). This means that the albedo decreased by 7 × 10^–3^ when the percentage of forest increased by 10%. The albedo level corresponding to the y-intercept was equal to the one previously observed on the target zone of pure natural grassland plotted in Fig. 4b. This local albedo around the stations, which is assumed to be slightly constant over the observation period 2000–2011, played a significant role in warming trends. For t_x_, this warming trend increased linearly with WSA with a slope of 0.385 ± 0.182 °C year^−1^ albedo unit^−1^ and a y-intercept of 0.063 ± 0.027 °C year^−1^ (R^2^ = 0.15; p-value = 0.04) (Fig. 5). This means that transition from natural grasslands with an albedo of 0.185 to a forest with an albedo of 0.125 dampened the warming trends by 0.23 °C per decade. For t_n_, no significant relationship between warming trend and WSA was observed. The slope was lower than the one obtained for t_x_ trend, with a value of 0.189 ± 0.217 °C year^−1^ albedo unit^−1^ (R^2^ = 0.03; p-value = 0.39). The dampening effect of forests on the warming trend for minimum temperatures was less than for maximum temperatures. This result tends to demonstrate the likely effect of biophysical variables such as albedo on changes in the regional field of air temperatures. However, this site-specific result does not contribute in explaining the slightly decreasing, statistically non-significant, elevation-dependent warming of spring maximum temperatures.

### IBPM simulations

**Figure 6:**
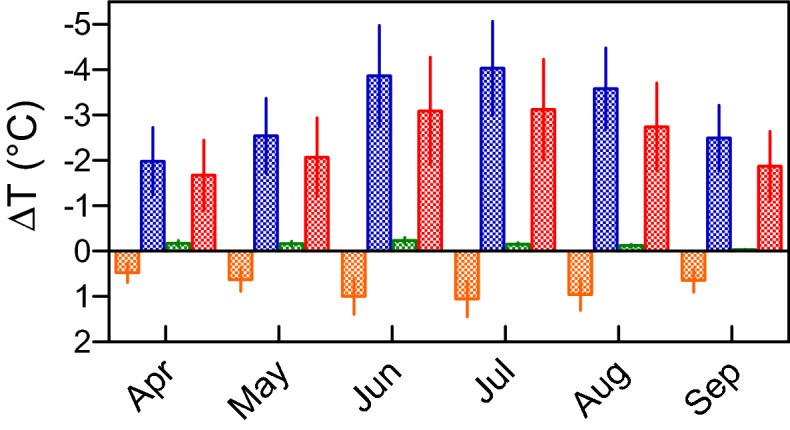
Components of monthly changes in surface temperature (red bar ± SD) in the afforestation scenario: shortwave radiative forcing effect due to albedo change (brown bar ± SD), energy redistribution associated with changes both roughness (blue bar ± SD) and Bowen ratio (green bar ± SD).

In our afforestation scenario, we applied the IBPM model. We examined the three components of change in surface temperature separately. In April, the applied albedos were respectively 0.180 ± 0.002 and 0.110 ± 0.006 for natural grasslands and forests. Those in July were 0.184 ± 0.002 and 0.124 ± 0.002. Over the 6-month period analyzed, changes in albedo were rather constant and weak, ranging from 0.059 ± 0.009 and 0.070 ± 0.003. As a consequence, changes in daily net shortwave radiation were limited ranging from + 24.6 ± 9.8 W m^−2^ in September to + 37.7 ± 10.1 W m^−2^ in July. The shortwave radiative forcing effect due to albedo change yielded a warming between + 0.48 ± 0.22 °C in April and + 1.06 ± 0.39 °C in July. The main effect was the result of changes in roughness. Figure SI4 plots the monthly field of wind velocity. Mean daily wind speed at 2-m height was the highest in April with a value of 2.64 ± 1.05 m s^−1^ and the lowest in August at 2.00 ± 0.75 m s^−1^. The corresponding aerodynamic resistances for grasslands and forests were respectively 38.5 ± 15.2 s m^−1^ and 2.5 ± 1.0 s m^−1^ for April and those for August, 44.8 s m^−1^ ± 19.2 and 2.9 ± 1.1 s m^−1^. Monthly cooling induced by changes in the energy redistribution factor attributable to changes in surface roughness ranged from − 1.98 ± 0.75 °C in April to − 4.03 ± 1.04 °C in July. The role of energy redistribution associated with changes in Bowen ratio was limited. Figure SI5 represents the time courses of Bowen ratios of both ecosystems. During the well-watered month of April, Bowen ratio for natural grassland was 1.79 ± 0.41 and 1.38 ± 0.15 for forest. With the occurrence of the summer drought, this ratio increased for both ecosystems respectively to 7.48 ± 1.09 and 5.25 ± 0.70 in July and then 7.10 ± 1.66 and 5.34 ± 0.79 in August. The Bowen ratio cooling only reached − 0.17 ± 0.07 °C in April and − 0.15 ± 0.04 °C in July. Finally, by summing up the three terms of the surface temperature change, we obtained a decrease between − 1.67 ± 0.78 °C in April and − 3.12 ± 1.11 °C in July (Fig. 6). We demonstrate here that the drivers of this unexpected spring warming are not local and surely not related to landscape changes, so we studied further atmospheric patterns known to have a significant impact on the Mediterranean climate.

### Large-scale influences

By considering Mont Aigoual as our reference station, we analyzed the correlation between the monthly temperatures t_x_ and t_n_ from 1980 to 2015 with the corresponding values of three teleconnection indices, i.e. WEMO, NAO and MO. The time courses of the nonparametric Spearman’s correlation have been reported on Table 2. For WEMO, Spearman r was highly significant (p < 0.001) in April and May for both t_x_ and t_n_. The correlation declined in June but with p-values remaining nevertheless < 0.01. The second time-period for which we observed a significant statistical link between WEMO and temperatures was from September to November with a peak in September (p < 0.001) followed by a gradual decrease in the significance. Correlations during the other months were not significant. For NAO index, highly significant correlations were observed for t_x_ in January, February and September those of March and May having p-values < 0.01. For t_n_ a high significance was only observed for February and September, January having a p-value < 0.05. With MO, only correlations for February were significant with p < 0.001 for t_x_ and p < 0.01 for t_n_.

**Table 2:** Nonparametric Spearman correlation r between monthly values of t_x_ and t_n_ and three teleconnection indices over 1980–2015: WEMO, NAO and MO.

	WEMO		NAO		MO	
	t_n_	t_x_	t_n_	t_x_	t_n_	t_x_
Jan	− 0.184	− 0.239	0.334*	0.538***	0.062	0.300
Feb	0.120	0.026	0.646***	0.677***	0.432**	0.523***
Mar	− 0.316	− 0.323	0.314	0.439**	0.207	0.292
Apr	–0.723***	–0.743***	− 0.022	0.026	− 0.118	− 0.197
May	–0.715***	–0.682***	0.297	0.449**	0.124	0.188
Jun	− 0.477**	− 0.472**	− 0.024	0.080	− 0.032	0.025
Jul	− 0.291	− 0.255	− 0.172	0.131	0.118	0.131
Aug	− 0.209	− 0.184	0.228	0.278	0.133	0.302
Sep	− 0.615***	− 0.584***	0.619***	0.575***	0.217	0.264
Oct	− 0.482**	− 0.381*	0.249	0.310	0.113	0.199
Nov	− 0.383*	− 0.376*	0.264	0.316	− 0.116	0.045
Dec	− 0.128	− 0.143	0.143	0.267	− 0.030	0.092

Significant values are in bold with their significant levels: ***p < 0.001, **p < 0.01 and *p < 0.05).

By averaging indices and temperatures for April to June, months with highly significant warming trends (see Fig. 1), we found, as expected, highly significant correlations of WEMO with both t_n_ (r = − 0.618, p < 0.001) and t_x_ (r = − 0.698, p < 0.001). For the same period, the correlations were not significant for either t_n_ or t_x_ both with NAO and MO. For extending such analysis to the whole-data set, which is the 32 station data, we performed a principal component analysis (PCA). The first component of the PCA accounted for 43.7% and 32.8% respectively of the total variance for t_x_ and t_n_, and the second component only described 6.4% and 7.6%. The Spearman correlations between the three indices and the first eigenvectors were only significant for WEMO with r = − 0.54 (p < 0.001) and r = − 0.67 (p < 0.001) for t_n_ et t_x_ respectively. The link between climate indices and more specifically the WEMO and maximum and minimum temperatures in spring raises questions about the underlying mechanisms.

## Discussion

We have highlighted ongoing warming trends that are largely higher than those observed at global scale for the same time step and for very close change points. For the three-month spring period from April to June, the warming trend reached for maximum air temperature 0.124 °C year^−1^, i.e. 1.24 °C per decade. This warming was uniform whatever the altitude. For the same period, minimum air temperature increased by 0.058 °C year^−1^ at 500 m altitude and this warming rate reaches 0.089 °C year^−1^ at 1500 m. All other months displayed the weakest warming trends which were not statistically significant. For comparison, global rates over 1979–2010 ranged from 0.141 ± 0.015 to 0.175 ± 0.012 °C/decade depending on data sources^1^.

Our results agreed with previous reported warming rates in the north western Mediterranean basin. They appeared to be the highest across the world and contributed to place the Mediterranean region as a climate change hot-spot^27^. Over Spain, the warming rate is particularly significant for mean air temperature in spring and summer and close for both seasons to 0.6 °C/decade^12^. However, here exist regional variations. In Catalonia (Spain), Reference^28^ reported only significant warming rates in spring with similar magnitude to our observations. Over Italy, Reference^29^ observed a peak trend occurring in summer with mean air temperature increasing from ca. 0.65 °C/decade. While a consensus is emerging that fall and winter are not significantly affected by warming, the other two seasons are those with the highest elevated warming rates. However, the fact that either spring or summer or both may be affected suggests that underlying meso-scale climate processes are active. If we shift our focus to mid-altitude mountainous regions, the scarcity of observations does not allow us to generalize our results. However, sparse evidences do indicate an increase in the warming rates with altitude, such as that observed more often in high mountains^10^. In Spain, Reference^15^ observed it in the Montseny natural park (Catalan Pre-Coastal Range) and^16^ in the Sierra de Cazorla (Betic mountain range). Similarly, Reference^30^ noted that the increase in air temperature is higher with altitude in the Abruzzo Region (central Italy).

Among the causes of enhanced warming of such magnitudes, the degree of landscape changes should be considered. Land Use and Land Cover (LULC) change is an important driver of environmental change, occurring at the same time as, and interacting with, climate change^31,32^. In the Cévennes National Park, we observed a rapid spreading of forests and woodlands at the expense of rangelands. Such changes have led to a linear increase in the percentage of forest cover from 38 to 68% between 1955 and 2015, at the expense of non-forested areas which declined from 62 to 25%. This landscape closure occurred in parallel with an increase in percentage of coniferous forests within the forest land cover type. In our study area, mechanisms of invasion by *Pinus* spp. have been detailed in^33^. These mechanisms have a rather broad nature^34^. Afforestation has already led to a significant decrease in water yield flowing from catchments whose headwaters are located within the CNP^35^ and significant losses of biodiversity^36^. During the last centuries, Mediterranean mountain landscapes have experienced a gradually decreasing intensity of human impacts due to declines of small-scale agriculture, rangelands, and forest use, particularly in low productivity areas^20,37,38^. The increasing abandonment of mountain and rural areas, generally triggered by the decline of livestock grazing induced a natural expansion of tree cover arising from secondary succession or closure of pre-existing woodlands. Natural reforestation is a heterogeneous and site-dependent process that is driven by topographic, climate, and socio-economic factors^33,39^. So, we identified common mechanisms in the CNP, in Spain^19,40^ and in Italy^41^.

Recently, attention has focused on understanding the potential for LULC change to affect surface temperature by modifying biophysical properties, energy acquisition and partitioning^42^. In this work, we limited our analysis in assessing consequences of changing vegetation cover with the highest contrast: natural grasslands and dense coniferous forests. Surface temperature is a state variable reflecting land surface–atmosphere feedback driven by water and energy exchange, and it is linked to the roughness of vegetation cover and the water exchange processes, which have been synthesized here by the Bowen ratio. As a result of our simulations, forests with a lower albedo absorb more solar irradiation, leading to a warming component ranging from 0.5 to 1.1 °C from April to July when compared to nearby grassland. The enhanced turbulent fluxes of latent and sensible heat of forest have a combined cooling effect, from − 2.2 to − 4.2 °C that offsets the albedo warming effect. Finally, at monthly time scale, the surface of the forest is 1.7–3.1 °C cooler than the grassland, indicating a substantial cooling effect. Our results are close to observations obtained in comparing paired flux towers^32,42^ or from remotely-sensed products^43^. However, assessing climate mitigation potential of forests requires an understanding of how LULC change affects not only surface temperature but also air temperature. To this point, extending effects of surface temperature to effects on air above the surface has been difficult^44^. For this reason, apparently puzzling results of warmer surfaces under colder air and vice versa have been reported^45^. This paradox extends to regional scale assessments^46^. The impact of a surface forcing is determined by the effective heat capacity of the atmosphere, which is defined by the depth of the planetary boundary layer. This can vary by an order of magnitude on contrasted time and spatial scales, and so this results in a much boosted temperature response in shallow boundary layers compared to a thicker one. Over mountainous terrain, multi-scale thermally driven flows play a key role in developing the planetary boundary layer and understanding of the underlying processes are still limited^47^. Therefore, it is difficult to infer consequences in terms of air temperature trends of the large LULC changes that drastically affect the CNP landscapes. However, the analysis we conducted in mapping the albedo surrounding the meteorological stations of our study area gives us a reasonable answer. The decrease in albedo from 0.185 to 0.125, i.e. the one corresponding to the change from natural grasslands to dense coniferous forests, translates into a cooling of the maximum temperatures of 0.26 °C/decade which contributes to slow down the warming rate enhancement. This result is completely in line with the estimates given in the synthesis of^48^.

As we demonstrated that the drivers of this unexpected spring warming are not local and surely not related to landscape changes, we studied three climate indices known to have significant impact on the Mediterranean climate: the North-Atlantic Oscillation (NAO), the Mediterranean Oscillation index (MO) and the Western Mediterranean Oscillation index (WeMO). Difficulties in the interpretation arise in using climate indices to the fact that another level of complexity is added, namely the link between climate indices and local climate. Several studies have shown that climate indices are linked with precipitation regimes and temperature trends^24,29^. European climates are particularly influenced by NAO, a north–south sea-level pressure dipolar pattern, with one of the centers located over Iceland and the other one approximately over the Azores Islands that controls the location and strength of storm tracks over the North-Atlantic and drives patterns in temperature and precipitation. Over the last decades, the phase of the NAO has been shifting from mostly negative to mostly positive index values^49^. As a consequence, this results in an intensified westerly ¯flow during the positive phase, that brings warm maritime air to Europe leading to a warming of southern Europe. Reference^50^ observed “the similarity of the departure patterns of temperature anomalies over these regions are strongly related to the persistent and exceptionally strong positive phase of the NAO index since the early 1980s”. However, in our study its impact remains rather limited, particularly with respect to spring temperatures. Other teleconnections are known to also significantly influence the Mediterranean climate, particularly the WeMO index. A positive phase of the WeMO typically shows an anticyclone located in the Gulf of Cádiz and a low-pressure area in Genoa Gulf whereas a negative WeMO phase will show a low in the Gulf of Cádiz and an anticyclone in Central Europe. During the positive phase, the prevailing winds are typically west and northwest, originating from the North Atlantic area. In contrast, a negative WeMO phase is associated to airflow which has traveled over the Mediterranean Sea. This configuration is at the origin of the high spring warming that we have evidenced in the Cevennes National Park. WeMO has been retained as a proxy for local environmental conditions such as Sea Surface Temperature (SST) with negative values associated with high SST^51^.

The evolution of near-surface air temperature is mostly impacted by radiation, heat advection, and land–atmosphere interactions. Refernce^52^ demonstrated that the larger part of year-to-year variation and long-term trend of daily temperature can be explained by the radiation. The effects of incoming shortwave and thermal longwave radiations have to be analyzed apart. A widespread reduction of incoming shortwave or global solar radiation between the 1950s and 1980s has been observed and since late 1980s a reversal in this trend has been recorded in many regions of the world^53,54^. Changes in transmissivity of the atmosphere due to change in anthropogenic aerosol emissions and cloudiness are considered to be the most likely causes^53,55,56^. For mainland Europe, daily brightening may reach 4.5 W m^−2^ per decade with a significant amplification in spring for the southern regions^57,58^. For the north western Mediterranean basin, this seasonality covariates with large-scale atmospheric circulation^59^. Brightening contributes in part to the rapid temperature increase since 1980 we observed over the CNP and that is considerably larger than the rise expected from greenhouse gas increases alone^60^.

The negative WeMO spring phases are associated with a strong east to west positive gradient in atmospheric humidity caused by evaporation as air travels across the Mediterranean Sea, leading to a strong water vapor feedback enhancing the forcing and temperature rise by about a factor of three^61,62^. Downward longwave radiation increases in response to increasing air specific humidity, i.e. the concentration of water vapor expressed as the mass of water vapor per unit mass of moist air. It is proportional to the partial pressure of water vapor and inversely proportional to the atmospheric pressure. This relationship is nonlinear with substantially higher sensitivities at low levels of air specific humidity, especially below 5 g kg^–1^^63^**.** These conditions occur more frequently during the cool spring season in the CNP area and might cause the observed elevation-dependent warmings^7^. Here, we suspect that additional heat release from water vapor acts as the main amplifying mechanism for climate change. This influence should be carefully studied in the future.

We have seen that air and sea interact on a wide range of scales, shaping climate and influencing weather. Coupled regional climate system models can help us to unravel these complex interactions. Recent simulations show that the atmospheric components correctly reproduce both large-scale and local features of the Mediterranean climate, although large temperature biases were observed. The sea component correctly reproduces the sea surface properties along with their interannual variation and circulations^64^. In spring, it is likely the air-sea interactions in the Mediterranean Sea that exert a significant influence on the regional biases^65^. Despite ongoing model improvements, it is suggested that higher spatial resolution could be needed for improving simulation of large-scale atmospheric circulations that impact the climate of the Cévennes National Park.

## Material and methods

### Regional setting

The study area is the Cévennes National Park (CNP further in the text). It is located in the natural region of the Cévennes, at the southern part of Massif Central Mountains in southern France. The park has two nested zones: a central protected zone, which has an area of 935 km^2^, and an adhesion or periphery zone, which covers 2785 km^2^. This transition space between the Massif Central and the Languedoc lowlands has specific characteristics. The park's altitude varies from 378 to 1699 m: highest elevations are located at Mont Lozère (1699 m) and Mont Aigoual (1567 m). Surface water from the Northern part of an axis from Mont Lozère to Mont Aigoual contributes to the Garonne watershed flowing to the Atlantic Ocean and the Southern part to the Rhône watershed flowing to the Mediterranean Sea.

The southern part corresponds to a Mediterranean-type climate turning gradually into a continental climate as it moves northward, leading to contrasted weather patterns. Its topography results in precipitation yearly totals ranging from 800 mm to almost 1800 mm. From 1901 to 2014, Mont Aigoual received an averaged amount of 1792 mm with 25% and 75% percentiles of 904 mm and 2117 mm. Annual average temperature over the whole-area ranged from 3 to 16 °C. Landscapes were shaped until the middle of the twentieth century by agriculture and pastoralism. Since 1950, the region has been subjected to a severe human depopulation, which has led to a severe agricultural decline and drastic changes in the landscape. Today the mosaic of vegetation units is more complex with coniferous forests (*Pinus sylvestris*, *P. nigra*), broadleaf deciduous (*Fagus sylvatica*, *Castanea sativa*) and evergreen (*Quercus ilex*) forests but also shrublands and grasslands.

### Climate analysis

Trends in monthly averaged daily minimum and maximum temperature data collected at 32 meteorological surface stations (henceforth stations) were analyzed with a non-parametric statistics, the Theil–Sen estimator^66^ (Sen 1968), more accurate than simple linear regression for skewed or heteroscedastic data. Data were downloaded from the MétéoFrance National Climatic Data Center (https://publitheque.meteo.fr). Stations were selected to maximize completeness, minimize station changes, and optimize spatial coverage over Cévennes National Park (central protected plus adhesion zones) and its close surrounding. We didn’t retain stations near or in urban areas to minimize the effects of urbanization on temperature.

Selected stations had at least 90% of data actually registered for the analysis period of 35 years, from January 1980 to December 2015 (see station list on Table SI1 and map on Fig. SI1). Each station was characterized by its elevation and aspect: north- or south-facing slope. Analyses were performed on time series of individual months for each station to determine if trends occurred during particular seasons. We choose the highest instrumented station, Mont Aigoual, as a reference site thanks to more than a century of monthly data. Additionally, we analyzed co-variations of minimum and maximum temperatures with monthly rainfall data from 13 stations among the 32.

### Vegetation mapping

The oldest map is based on visual interpretation of panchromatic orthorectified aerial photos (1:5000 scale) obtained from 1955 to 1960 and then published as paper map by the French National Geography Institute at 1/100,000 scale (NGI 1955).

The CORINE Land Cover maps were produced in 2000 (CLC2000) and in 2006 (CLC2006) as part of a European Community program to generate digital land-use/land-cover maps covering the European continent (https://land.copernicus.eu/pan-european/corine-land-cover). The maps were produced using satellite Landsat7 ETM + data and other ancillary data (Digital Elevation Model and aerial photos). The maps have 44 classes and a spatial detail comparable to that of the paper map or a 1/100,000.

Cevennes National Park (further CNP) developed its own digital maps for 1974 (CNP1974), 2000 (CNP000) and 2010 (CNP2010) by interpreting false color orthorectified aerial photos (1/5,000 scale) and ground truth sampling. Vegetation description included both horizontal (foliage projected cover) and vertical (canopy layering) attributes. CNP2010 incorporated floristic attributes: the dominant species for overstorey and understorey layers. Spatial details correspond to 1/50,000 scale.

Additionally, we used two other digital maps: the National Forest Inventory map for 1999 (NFI1999) was based on a regular 1 km × 1 km samples with detailed vegetation inventory on each grid point scaled up with the help of false color orthorectified aerial photos (scale 1/100,000) and the GISLR2006 built for the Occitanie region (where CNP belongs to) from supervised classification of satellite Landsat7 ETM + data (scale 1/100,000) available at OpenIG: https://www.siglr.org. For further analysis we grouped together vegetation classes in three major types: the forests type with dense and mid dense forests which combines broad-leaved, coniferous and mixed forests, the transition type with open forest and transitional shrub/woodland, and an ‘open’ type that includes low shrublands, heaths and moors, natural grasslands, pastures and areas principally occupied by agriculture. Urban structures are not considered here.

### Surface albedo

In order to characterize regional pattern of albedo changes, we used MODIS (Moderate Resolution Imaging Spectrometry) MCD43A3 albedo products (Collection 5) available at the geoportal https://lpdaac.usgs.gov/data_access. Albedo products are derived from surface reflectances, corrected for atmospheric scattering, ozone absorption, and aerosols. They represent an eight-day 500-m composite combining reflectance data from Terra and Aqua satellites. As albedo depends not only on surface properties, but also on solar angle and atmospheric conditions, we considered in this work white-sky albedo to isolate the dependence of albedo on surface characteristics. White-sky albedo (WSA) or diffuse albedo, represents bi-hemispherical reflectance under isotropic skylight illumination; therefore, it is independent of sky conditions. WSA is particularly useful for characterizing biophysical variables of contrasting land covers at pixel scales. They were used primarily to evaluate differences in surface albedo between contrasted vegetation types over 12 to 16 pixels for target zones of pure vegetation class relatively unchanged from 2000 to 2011. We also evaluated the mean surface albedo of area of 36 pixels centered on the 32 weather stations concurrently with the percent cover of the ‘forest’ type.

### Change in surface temperature with the intrinsic biophysical mechanism model

We limited this analysis in assessing consequences of changing vegetation cover from natural grasslands to dense coniferous forests on the energy balance and consequently, on surface temperature. For this purpose, we applied the intrinsic biophysical mechanism (IBPM) model proposed by^31^ in prognostic mode (details are available in the supporting information). The change in land surface temperature depends on three terms: (1) the shortwave radiative forcing effect due to albedo change and the role of energy redistribution associated with changes in both (2) roughness and (3) Bowen ratio. We focused here only on monthly averaged daily temperature for the period ranging from April to September. The daily climate data came from the Lafage-INRA experimental station (lat. 3°05′E, long. 43°55′N, 804 m asl, see Table SI1): maximum and minimum temperatures, precipitation, global radiation and wind speed. Albedo variations are derived from MODIS WSA (see previous paragraph). The aerodynamic resistances (*r*_a_) of the two vegetation covers (grassland and coniferous forest) are calculated monthly for a wind velocity which is the average velocity observed for each month of the selected time window (see Fig. SI3). The wind speed profile with height is assumed logarithmic. Vegetation heights *h* are 0.2 m and 15 m respectively for the two vegetation types. Aerodynamic resistances are estimated with the momentum equation:

$${r}_{a}={\frac{1}{{k}^{2}u}/\left[ln\left(\frac{z-d}{{z}_{0}}\right)\right]}^{2}$$

in which *k* is the von Karman’s constant = 0.41, *d* the zero-plane displacement = 0.67 h, *z*_0_ the roughness length = 0.13 h and *u* the wind velocity (m s^−1^) measured at height *z*.

The Bowen ratios *β *are obtained by simulating a simple soil water balance model based on the calculation of the Priestley–Taylor potential evapotranspiration (PET) and a relationship between the ratio of actual evapotranspiration (AET) on PET and the relative soil water content proposed by^68^. This model has been validated for natural grasslands on measurements of soil water storage (see^33^ and Fig. SI2). For coniferous forests, rooting depth is assumed to reach 1 m. The soil water retention properties remain unchanged. Maximum AET values of the Linacre equation has been those proposed by^67^.

### Large-scale influences: the role of teleconnection

The relationship between teleconnection patterns and air temperature was studied by means of the Pearson’s correlation coefficient. The selected teleconnection patterns were the most prominent for Western Europe, the NAO, and two Mediterranean patterns: the MO and the WeMO. The North Atlantic Oscillation index (NAO) values are from the Gibraltar—Iceland dipole^69^. The Mediterranean Oscillation index (MO) values are defined by the dipole Gibraltar—Lod Airport (Israel)^70^. Lastly, the Western Mediterranean Oscillation index (WeMO) has recently been suggested for South Western Europe^71^ using the dipole Cadiz (southern Spain)—Padua (central Italy), with the Mediterranean coastland as main area of influence. In their dipoles, the three indices share values of Gibraltar or its surrounding areas. The three indices were calculated using normalized sea level pressure. Daily MO and monthly NAO index were obtained from the Climate Research Unit, East Angelia University, UK (http://www.cru.uea.ac.uk/). MO data were further aggregated on a monthly time step. The WeMO monthly data were made available by the Climatology Group, University of Barcelona (http://www.ub.es/). The Pearson correlation coefficient has been used to quantify the relationships between large-scale circulations and both seasonal temperature time series and main eigenvectors of a Principal Component Analysis (PCA) processed on the whole-CNP t_x_ and t_n_ data set.

## Supplementary Information

Supplementary Information.
