# Supplementary material for: Enhanced spring warming in a Mediterranean mountain by atmospheric circulation
Source: Sci Rep. 2022 May 11;12:7721. doi: 10.1038/s41598-022-11837-x (PMC9095602; doi:10.1038/s41598-022-11837-x)

SUPPORTING INFORMATION

ENHANCED SPRING WARMING IN A MEDITERRANEAN MOUNTAIN BY ATMOSPHERIC CIRCULATION

Bruley E.[1,3], Mouillot F.[2], Lauvaux T.[4] & Rambal S.[1,5]

1 Centre d'Ecologie Fonctionnelle et Evolutive CEFE, UMR5175, CNRS, Université de Montpellier, Université Paul-Valéry Montpellier, EPHE, 1919 Route de Mende, 34293 Montpellier Cedex 5, France

2 Centre d'Ecologie Fonctionnelle et Evolutive CEFE, UMR 5175, CNRS, Université de Montpellier, Université Paul-Valéry Montpellier, EPHE, IRD, 1919 Route de Mende, 34293 Montpellier Cedex 5, France

3 Laboratoire d'Ecologie Alpine, CNRS, Université Grenoble Alpes, Grenoble, France

4 Laboratoire des Sciences du Climat et de l'Environnement, IPSL, Univ. de Saclay, Saclay, France

5 Universidade Federal de Lavras, Departamento de Biologia, CP 3037, CEP 37200-000, Lavras, MG, Brazil

The intrinsic biophysical mechanism model

The intrinsic biophysical mechanism model is based on the surface energy balance:

$$R_n = S + L_{\downarrow} - \varepsilon\sigma T_s^4 = H + G \qquad (1)$$

Where R_n is net surface radiation, S is net surface shortwave radiation, $L_{\downarrow}$ is incoming long wave radiation, ε is surface emissivity, σ is the Stephan-Boltzmann constant, T_S is surface temperature, H is sensible heat flux, LE is latent heat flux, and G is ground heat flux. Sensible heat is defined from the gradient between surface and air temperature and given as:

$$H = \rho C_p \frac{T_s - T_a}{r_a} \quad (2)$$

where ρ is air density, C_P is specific heat of air at constant pressure, r_a is aerodynamic resistance

$$H / \beta \qquad (3)$$

Latent heat is expressed as the ratio of sensible heat flux and Bowen ratio. Surface outgoing long wave radiation in (1) can be approximated using a Taylor series expansion with T_a:

$$T_s^4 = T_a^4 + 4\,T_a^4 (T_S - T_a) \quad (4)$$

Linearizing the surface longwave radiation term in Equation (1) and making use of Equations (2) and (3), we obtain a solution for T_s from Equation 4.

$$T_s = \frac{\lambda_0}{1+f}\left(R_n^{\square} - G\right) + T_a \qquad (5)$$

where R_n* is an apparent net radiation given by:

$$R_n^{\square} = S + L_{\downarrow} - \sigma T_a^4$$

and the energy redistribution factor f is given by:

$$f = \frac{\rho C_p}{4\sigma T_s^3 r_a}\left(1 + \frac{1}{\beta}\right) \qquad (6)$$

IBPM assumes nearby contrasting land covers share the same atmospheric background, such as air temperature and incoming radiation. Ignoring changes in surface emissivity and ground heat flux, surface temperature change ΔT_s can be derived by the first derivative of (5):

$$\Delta T_s = \frac{\lambda_0}{1+f}\Delta S + \frac{-\lambda_0}{(1+f)^2} R_n \Delta f \; (7)$$

$$\Delta f = \Delta f_1 + \Delta f_2$$

With $\Delta f_1 = \frac{-\rho C_P}{4\sigma T_S^3 r_a}\left(1 + \frac{1}{\beta}\right)\frac{\Delta r_a}{r_a}$ and $\Delta f_2 = \frac{-\rho C_P}{4\sigma T_S^3 r_a}\left(\frac{\Delta\beta}{\beta^2}\right)$

$$\Delta T_S \approx \frac{\lambda_0}{1+f}\Delta S + \frac{-\lambda_0}{(1+f)^2} R_n \Delta f_1 + \frac{-\lambda_0}{(1+f)^2} R_n \Delta f_2$$

where ΔS is the change of net shortwave radiation, and Δf is the change in the energy redistribution factor, attributable to changes in surface roughness (Δf_1) and Bowen ratio (Δf_2).

Table SI1. Temperature analysis: weather station location, longitude, latitude, elevation expressed in m asl and record length. We also reported aspects.

	Weather station	Long.	Lat.	Elev.	Record length	Aspec t
1	Arphy	3°35'E	44°01'N	427	1989-2015	South
2	Brenoux-Mende	3°32'E	44°31'N	1019	1980-2015	North
3	Cardet	4°05'E	44°01'N	109	1980-2015	South
4	Châteauneuf de Randon	3°41'E	44°38'N	1238	1980-2015	Nord
5	Florac	3°35'E	44°19'N	595	1980-2015	Nord
6	Générargues	3°59'E	44°05'N	139	1980-2015	South
7	Génolhac	3°58'E	44°21'N	519	1980-2015	South
8	Grospierres	4°18'E	44°25'N	125	1980-2015	South
9	Hures-la-Parade	3°24'E	44°15'N	1028	1983-2015	Nord
10	Joyeuse	4°14'E	44°29'N	212	1980-2015	South
11	La Fage-INRA	3°05'E	43°55'N	804	1980-2015	South
12	La Grand Combe	4°01'E	44°14'N	415	1980-2015	South
13	Lablachère	4°13'E	44°29'N	300	1980-2015	South
14	Langogne-Naussac	3°52'E	44°44'N	934	1980-2015	Nord
15	Le Caylar	3°20'E	43°52'N	730	1980-2015	South
16	Le Collet de Dèze	3°56'E	44°16'N	485	1990-2015	South
17	Le Massegros	3°10'E	44°18'N	873	1980-2015	Nord
18	Le Vigan	3°37'E	43°59'N	222	1980-2015	South
19	Loubaresse	4°03'E	44°36'N	1220	1980-2015	South
20	Mandagout	3°38'E	44°01'N	485	1983-2015	South
21	Mende Ville	3°30'E	44°31'N	713	1980-2015	Nord
22	Meyrueis	3°26'E	44°11'N	1054	1980-2015	Nord
23	Millau	3°01'E	44°07'N	712	1980-2015	South
24	Mont Aigoual	3°35'E	44°07'N	1567	1980-2015	South
25	Nasbinals	3°03'E	44°40'N	1284	1980-2015	Nord
26	Pont de Montvert	3°44'E	44°22'N	875	1980-2015	Nord
27	Prades le Lez	3°52'E	43°43'N	85	1980-2015	South
28	Saint Christol les Alès	4°05'E	44°05'N	129	1980-2015	South
29	Saint Jean du Gard	3°53'E	44°07'N	220	1980-2015	South
30	Saint Martin de Londres	3°44'E	43°47'N	194	1980-2015	South
31	Sumène	3°43'E	43°59'N	198	1980-2015	South
32	Villefort	4°00'E	44°26'N	620	1980-2015	Nord

Missing data: station number and year: 7 (2008); 14 (1997&1998); 27 (1991).

Table SI2. Rainfall analysis: weather station locations, longitudes, latitudes, elevations expressed in m asl and record length. We also reported aspects.

Weather station	Longitude	Latitude	Elevation	Record length	Aspect
Châteauneuf-de-Randon	3°41' E	44°38' N	1238	1901-2015	Nord
Florac	3°35' E	44°19' N	595	1901-2015	Nord
Generargues	3°59' E	44°05’ N	139	1901-2015	South
Génolhac	3°58' E	44°21' N	519	1901-2015	South
La Grand-Combe	4°01' E	44°14' N	415	1901-2015	South
Le Collet-de-Deze	3°56' E	44°16' N	485	1901-2015	South
Le Massegros	3°10' E	44°18' N	873	1901-2015	Nord
Mende	3°30' E	44°31' N	713	1901-2015	Nord
Meyrueis	3°26' E	44°11' N	1054	1901-2015	Nord
Mont Aigoual	3°35' E	44°07' N	1567	1901-2015	South
Nasbinals	3°03' E	44°40' N	1284	1901-2015	Nord
Pont-de-Montvert	3°44' E	44°22' N	875	1901-2015	Nord
Saint-Jean-du-Gard	3°53' E	44°07' N	220	1901-2015	South

Fig SI1. The Cévennes National Park (grey area): the park has two nested zones: a central protected zone bounded by the green line, which has an area of 935 km^2, and an adhesion or periphery zone, which covers 2785 km^2. The weather stations are indicated by red dots.

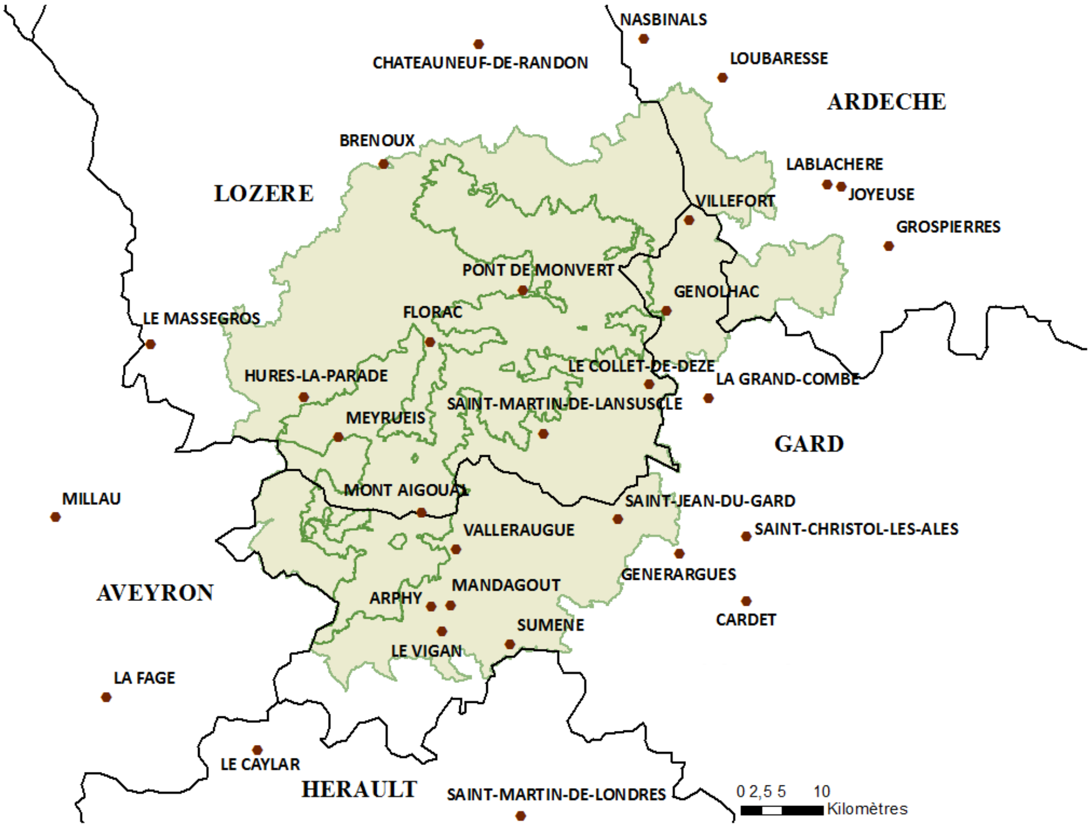

Fig SI2 Simulated soil water storage in the upper 40 cm (plain line) and observed data (circles with bars showing standard deviation) from March to October in 2005 and 2006. Observed measurements include data from at least 10 pairs of time domain reflectometry (TDR) probes set up in four natural grasslands. Daily rainfall amounts are shown at the bottom (see Bouland et al. 2008).

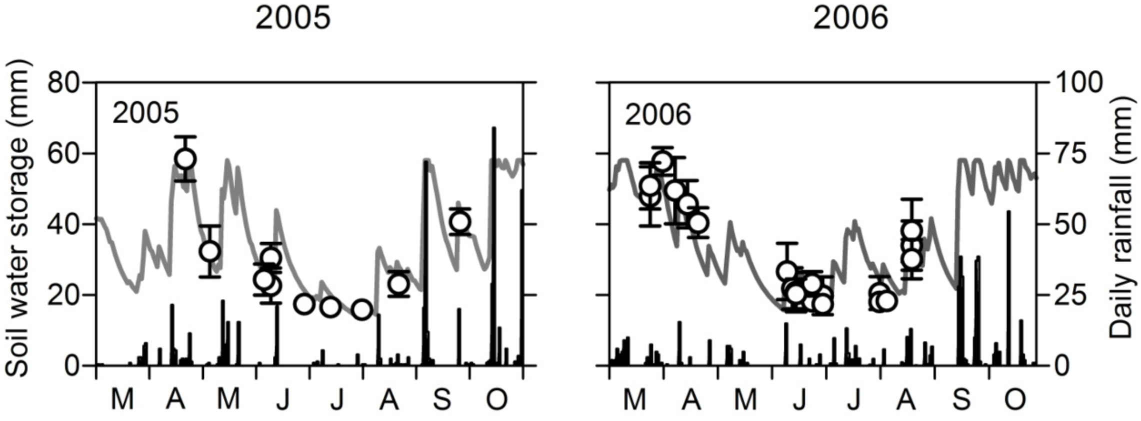

Figure SI3 Time courses of yearly (blue line) and April to June (red line) rainfall amount in two contrasted weather stations of the study area: the driest, Générargues and the wettest, Mont Aigoual. Dashed lines are for the non significant linear trends.

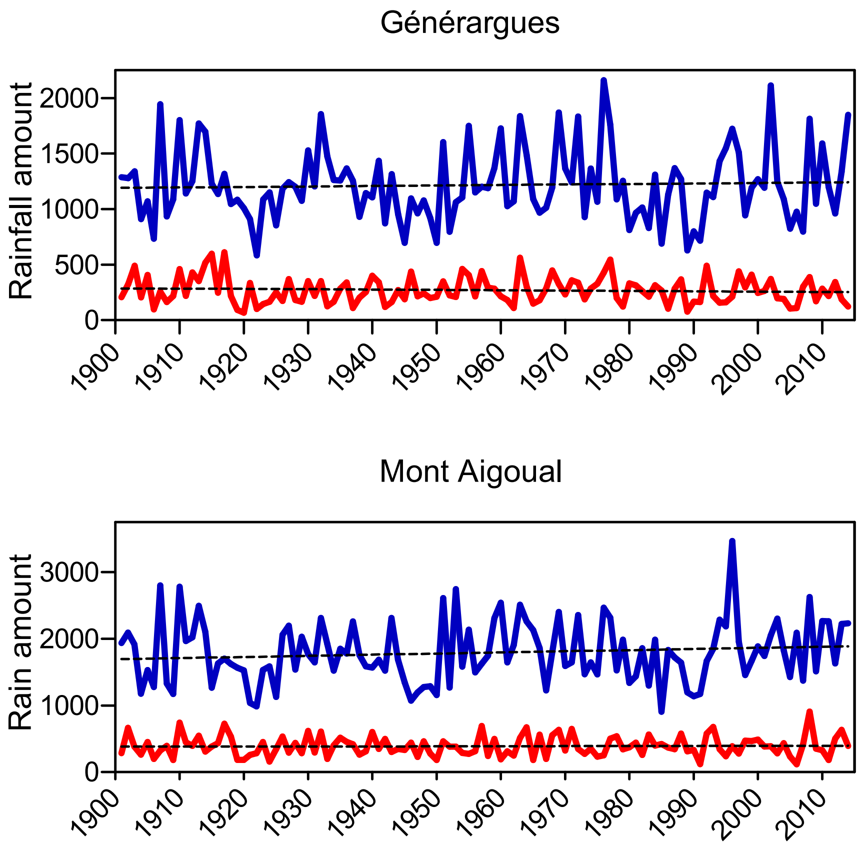

Fig SI4. Daily wind velocity by month over 2008-2014 observed at Lafage-INRA experimental station (lat. 3°05'E, long. 43°55'N, 804 m asl). Red lines are for averaged values.

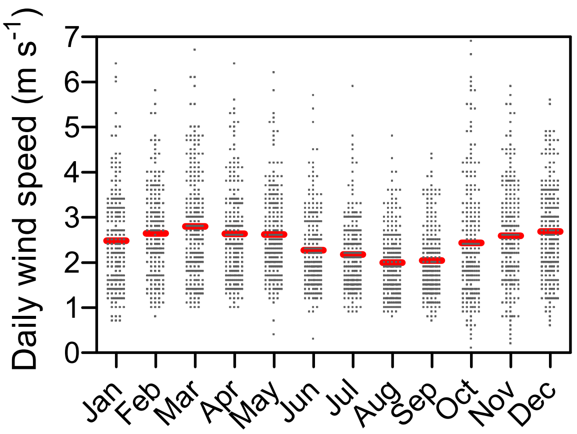

Fig SI5 Time courses of the Bowen ratios averaged over 2005-2015 for grassland (light green filled circle ± SD) and coniferous forest (dark green filled circle ± SD).

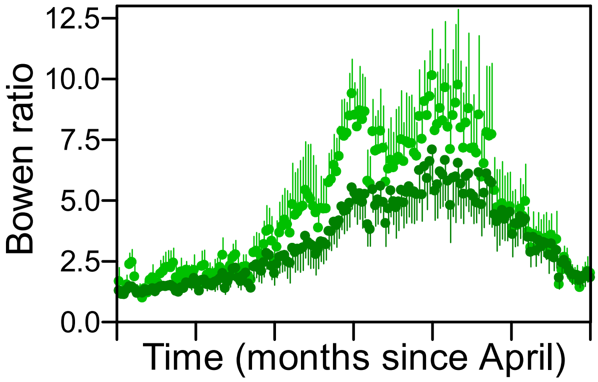

Supplement: Supplementary file 1 — Supplementary Information. [file 41598_2022_11837_MOESM1_ESM.pdf]
